# Biochemical and Transcriptomic Analysis Reveals Low Temperature-Driven Oxidative Stress in Pupal *Apis mellifera* Neural System

**DOI:** 10.3390/insects16030250

**Published:** 2025-03-01

**Authors:** Xiangjie Zhu, Mingjie Cao, Chenyang Li, Chenyu Zhu, Han Li, Yuanmingyue Tian, Jiaqi Shang, Jiaqi Sun, Bingfeng Zhou, Xianda Wu, Shujing Zhou, Xinjian Xu

**Affiliations:** 1 College of Bee Science and Biomedicine, Fujian Agriculture and Forestry University, Fuzhou 350002, China; xiangjie_zhu@126.com (X.Z.); jack-2001@live.cn (M.C.); zcy1775880093@163.com (C.Z.); 18686489310@163.com (H.L.); 1220644012@fafu.edu.cn (Y.T.); 12407044019@fafu.edu.cn (J.S.); 12407044018@fafu.edu.cn (J.S.); bingfengfz@126.com (B.Z.); 2 Honeybee Research Institute, Fujian Agriculture and Forestry University, Fuzhou 350002, China; 3 Center for Plant Metabolomics, Haixia Institute of Science and Technology, College of Horticulture, Fujian Agriculture and Forestry University, Fuzhou 350002, China; lichenyang425@163.com; 4 Academic Journal Department, Social Sciences Division, Fujian Agriculture and Forestry University, Fuzhou 350002, China; wuxianda405@163.com

**Keywords:** honeybee, cold stress, newly pupated stage, brain development, oxidative stress

## Abstract

Exposure to low temperatures during honeybee development has been shown to impede brain development and affect cognitive function in adult bees. On the other hand, neuronal damage due to oxidative stress has been reported in many cases. Hence, biochemical parameters related to oxidative stress in honeybee pupae brain were determined. The levels of GSH in the pupal brain decreased after 24 h and 48 h of exposure to low temperatures; there were also reduced activities of SOD and CAT enzymes following 48 h of low-temperature treatment compared to the control group. Furthermore, analysis of transcriptome data post-24 h and -48 h low-temperature stress revealed the suppression of the glutathione metabolism and peroxisome pathways in pupal brains. Additionally, expression pattern clustering analysis and KEGG enrichment showed that 10 differentially expressed genes with down-regulated expression trends post-low-temperature treatment were significantly enriched in the peroxisome pathway, including *PEX10*, highlighting their connection to peroxisome function. RT-qPCR validation was conducted on 11 core enriched genes in pathways identified via GSEA, and all these genes exhibited a downregulated expression pattern, confirming the inhibition of glutathione metabolism and peroxisome function under low-temperature stress. The present study showed that exposing honeybee pupae to low temperatures suppressed both the glutathione metabolism and peroxisome pathways, resulting in increased oxidative stress. This research enhances our understanding of how the pupal brain reacts to cold stress and illuminates the neural damage associated with low temperatures during honeybee capped brood development.

## Simple Summary

Honeybees, with their narrow developmental temperature range, serve as an ideal model for investigating temperature-induced insect development. Exposure to low temperatures during the pupal stage can lead to impaired learning, memory, and foraging abilities post-emergence. By detecting a number of oxidative stress-related parameters, it was demonstrated that low temperatures result in increased oxidative stress in the honeybee pupa brain. Furthermore, transcriptomic analyses revealed that the inhibition of glutathione metabolism and peroxisomal pathways at low temperatures may be responsible for the elevated oxidative stress, which may play a significant role in the developmental defects observed in the brains of pupae exposed to cold temperatures. This contributes to our understanding of honeybee brain dysfunction under suboptimal brood-rearing temperatures.

## 1. Introduction

The potential impact of temperature fluctuations resulting from global climate change on honeybees is a significant concern for their long-term survival [1]. The ability of the colony to regulate the hive temperature at 35 °C is vital for the survival of honeybees, as they require a narrow temperature range for their development across different climatic conditions [2]. However, substantial climatic shifts, such as disruptions to the seasonal cycle and unexpected temperature fluctuations, may adversely affect honeybee thermoregulation. This can lead to disruptions in hive temperature, impacting the homeostasis, growth, and survival of the colony [3]. In cases of spring cold spells, when only a few overwintering honeybees remain in the nest, the colony’s capacity to maintain internal temperature is limited. Additionally, honeybee broods located at the periphery of the brood-rearing area are vulnerable to prolonged low-temperature stress conditions. Consequently, the colony may take significantly longer to produce new honeybees or may even fail to generate enough to replace the existing population, resulting in colony loss. This issue has become a focal point in beekeeping practices.

In comparison to many other insects, honey bees have a much narrower developmental temperature range (32–36 °C; the optimum temperature is 35 °C) [4,5], and temperature deviation from the optimum range will result in increased honeybee mortality [6], developmental process [7], birth weight [8], or external morphology [5], but some showedstress resistance [9,10]. Specifically, honeybee capped broods exhibit developmental stagnation under low-temperature stress [11], affecting various aspects, including brain development [12]. The resumption of development occurs upon the restoration of appropriate temperatures, yet adult honeybees reared under constant lower temperatures display a diminished capacity for associative learning and memory [12]. Notably, honeybees raised in such conditions show morphophysiological alterations in the mushroom bodies and synaptic organization of their brains [4], potentially hindering foraging abilities and weakening the colony [13]. Exposure to low temperatures during honeybee brood rearing evidently leads to neural system deficiencies, although the precise regulatory mechanisms governing this process remain elusive.

The accumulation of reactive oxygen species (ROS) is hypothesized to play a crucial role in the pathogenesis of neurological damage in the honeybee brain. Research on the sublethal impacts of imidacloprid on honeybees suggests that the decreased expression of specific genes related to oxidative stress may contribute to heightened neural apoptosis [14]. Similarly, a study on the effects of tebuconazole exposure on honeybees demonstrated that disrupting redox homeostasis and modifying the fatty acid profile could potentially lead to neurotoxicity [15]. Furthermore, it has been suggested that alleviating impaired brain function in honeybees may be achieved through the suppression of gene expression associated with ROS accumulation, underscoring the significance of ROS accumulation in inducing neurological dysfunction in the brain [16].

Various stressors, such as low temperatures, can induce oxidative stress by increasing the production of ROS while impairing their scavenging systems [17]. We propose that oxidative stress plays a significant role in causing neurological hypoplasia in honeybee pupae. For this study, we specifically chose honeybee pupae in the immediate post-pupation stage, which displayed heightened sensitivity to low temperatures [11]. Our aim was to examine the changes in oxidative stress levels in the brains of honeybee pupae under low-temperature stress and to elucidate the related molecular pathways through biochemical and transcriptomic investigations. This research offers novel insights into the neural toxicity induced by low temperatures in honeybee developmental stages, thereby enhancing our understanding of this prevalent environmental challenge in apiculture and facilitating a more precise evaluation of the associated ecological risks.

## 2. Materials and Methods

### 2.1. Sample Preparation

The samples used in this study were collected from three different healthy colonies during the spring season, with no signs of known brood diseases. Using the sampling method previously developed by our research group to obtain consistent honeybee eggs [11], each queen was confined in a mesh cage to lay eggs on a fresh comb for a period of 12 h. The eggs were left in the colony until sealed by workers. Sections of the comb containing broods capped within 4 h were placed in a chamber with a controlled temperature and humidity (35 °C ± 0.2 °C, RH 75%) for 4 days (controls).

Low-temperature stress causes slow or stagnant brain growth of new pupae, resulting in significantly higher mortality and the deterioration of associative learning ability in adulthood, suggesting a potential cause of brain damage during developmental stagnation under low-temperature stress [12]. Based on this developmental characteristic, 24 h and 48 h as the gradient intensity of the low-temperature treatments were applied to the new pupae sample (namely T1, T2). Subsequently, two groups of capped broods were transferred to another chamber and subjected to low-temperature treatment (20 °C ± 0.2 °C, RH 75%) for 24 h and 48 h (low-temperature treated, T1 and T2). Pupae heads were collected by manual uncapping, with twenty heads per replicate immediately immersed in liquid nitrogen and stored at −80 °C for further analysis. Every biological replicate was sourced from an individual bee colony. Biochemical analysis was conducted on three replicates from each comparison group, and three replicates were allocated for RT-qPCR gene expression analysis.

### 2.2. Measurement of Oxidative Stress Parameters

Samples collected in Section 2.1 were frozen using liquid nitrogen and stored at −80 °C for the analysis of oxidative stress parameters. Each group was evaluated with three biological replicates and three technical replicates. Each biological replicate consisted of a pooled head tissue sample from 10 individual honeybee pupae. Protein concentrations were measured using a BCA Protein Assay Kit (MA0082, meilunbio, Dalian, China). The content of glutathione (GSH) was quantified using an enzymatic recycling method [18]. The superoxide dismutase (SOD) and catalase (CAT) activities were assayed using an SOD Activity Detection Kit (WST-8 method, G0101W) and CAT Assay Kit (G0105W). The detection kits for GSH content, SOD activity, and CAT activity were obtained from Grace Biotechnology (Suzhou, China). All measurements were carried out in accordance with the manufacturer’s guidelines.

### 2.3. Gathering and Processing of Transcriptomic Data

Transcriptomic data on the impact of varying degrees of cold stress on the heads of pupal *Apis mellifera* were retrieved from the Sequence Read Archive (SRA) database at the National Center for Biotechnology Information (NCBI), under project accession number PRJNA1221518, as previously collected by the research group of the authors [12]. We selected data from honeybee pupal heads without temperature treatment (control group, CK) and with 20 °C cold treatments for 24 h and 48 h (low-temperature treatment groups, T1 and T2) for analysis; each sample contained three biological replicates. The sequencing samples were processed identically to those used for oxidative stress index measurements, aiding in our exploration of the underlying mechanisms at the gene expression level.

The reference genome Amel _ HAv3.1 (NCBI Assembly: GCF_003254395. 2) was used for reconstituting the transcripts. The expression levels of the transcripts were determined using the FPKM (fragment per kilobase of transcript per million mapped reads) method, with StringTie being utilized for the computation of FPKM to quantify gene expression abundance [19]. The formula for calculating FPKM for a given transcript is as follows: FPKM = 106C/(NL*103) (C is the number of fragments mapped to the transcript, N is the total number of fragments mapped to the reference gene, and L is the number of bases on the transcript). Differentially expressed genes (DEGs) between the two samples’ treatments were analyzed using the DEGseq R package [20]. DEGs were identified using a Poisson distribution with a false discovery rate (FDR)  ≤  0.05 and fold change  ≥  2 (log2 ratio  ≥  1, fold change = FPKM of the treatment group/FPKM of the control group) through multiple hypothesis testing. Significant differential expressions were determined based on this threshold. Additionally, we used GSEA (Gene set enrichment analysis) to identify distinct pathways between the control and low-temperature-treated groups [21], setting a significance threshold of |normalized enrichment score |(NES)| > 1 and FDR < 0.05. GSEA result were produced online using the Omicsmart platform.

### 2.4. Clustering Analysis and Functional Classification

The DEGs in the control group (CK) and two treatment groups (T1, T2) were then clustered using the ClusterGVis package (Jun Zhang (2022), ClusterGVis: One-step to Cluster, and Visualize Gene Expression Matrix, https://github.com/junjunlab/ClusterGVis accessed on 10 October 2024). The curve depicting the changes in the sum of squared errors with respect to the number of clusters was derived from the expression matrix. Subsequently, the inflection points of the curve were utilized to determine the optimal number of clusters (Figure S1). The DEGs in each cluster were subjected to KEGG pathway enrichment analysis and hypothesis testing was performed to obtain P values; the threshold of *p* < 0.05 was used to determine the significance of pathway enrichment. Enrichment analysis was performed through OmicShare tools [22].

### 2.5. RT-qPCR Validation

The primers for this study were designed using SnapGene software (version 6.0.2) and are listed in Table S1. Each sample underwent three biological replicates and three technical replicates for the target gene. Three biological replicates were obtained from three different colonies, each pooled from the heads of three pupae per replicate. The total RNA was extracted from the samples gathered in the Section 2.1. using the Transzol^®^ Up Plus RNA Kit (TransGen Biotech, Beijing, China), and cDNA was synthesized through reverse transcription using TransScript^®^ Uni All-in-One First-Strand cDNA Synthesis SuperMix (TransGen Biotech, Beijing, China) for qPCR. The resulting cDNA was then used as a template for quantitative PCR (qPCR), with a reaction system of 10 μL following the PerfectStart^®^ Green qPCR SuperMix (TransGen Biotech, Beijing, China) instructions. The 2^−△△Ct^ method was used to calculate the relative expression level of the studied genes [23], with *actin* used as a reference. Finally, the expression level was logarithmically transformed using a base of 2, and the result was determined by the average. The difference in gene expression between the treatment group and the control group was analyzed using an unpaired *t*-test to determine statistical significance.

### 2.6. Data Analysis

Student’s *t*-test was used to assess the differences in gene expression levels and biochemical parameters between the low-temperature treatment groups and controls. Statistical significance was defined as *p* < 0.05 (*), *p* < 0.01 (**) and *p* < 0.001 (***).

## 3. Results

### 3.1. Pupal Development Arrest Post-Low-Temperature Treatment

**Figure 1:**
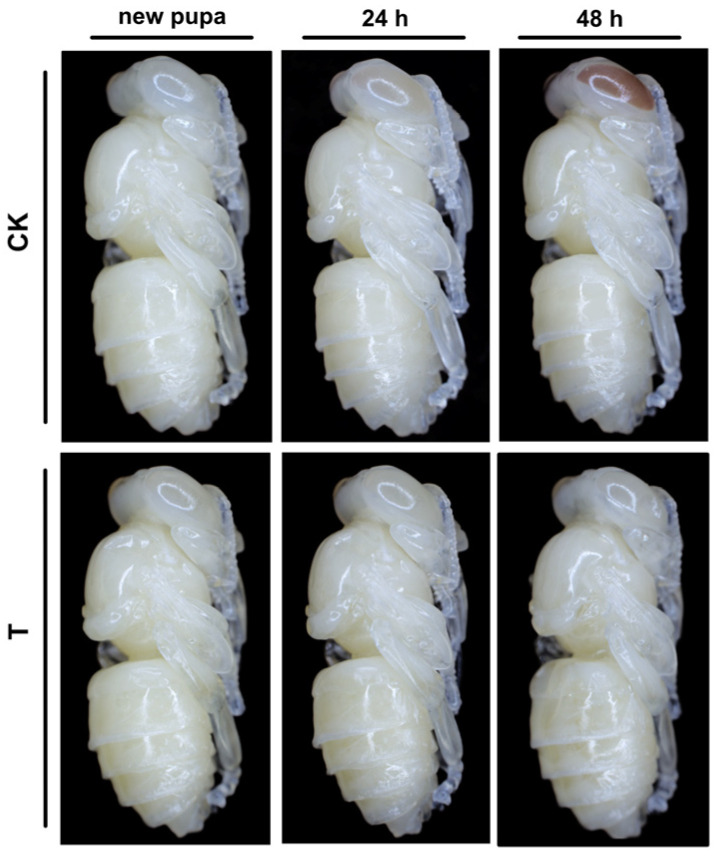
Effects of low-temperature stress on new pupae *Apis mellifera* development. CK, 35 °C control group; T, 20 °C low-temperature treatment; h, hour.

In this study, we recorded the appearance of honeybee new pupae under low-temperature exposure and found that they remained white-eyed after 24 h and 48 h of 20 °C low-temperature treatment and exhibited an overall developmental arrest, including the head region (Figure 1).

### 3.2. Oxidative Stress Parameters in Pupal Brains Post-Low-Temperature Treatment

**Figure 2:**
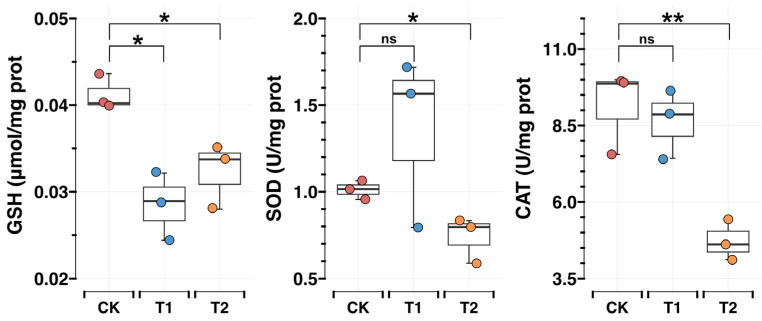
Effects of low-temperature stress on oxidative stress parameters in pupal brains *Apis mellifera*. Asterisks indicate significant differences between comparison group (* *p* < 0.05, ** *p* < 0.01, ns *p* > 0.05, Student’s *t*-test, T1, T2 denote 24 h, 48 h 20 °C low-temperature treatment, respectively).

The reduced glutathione is a reactive oxygen species (ROS) defender and is often considered as one of the markers for oxidative stress detection [24,25]. Superoxide dismutase (SOD) and catalase (CAT) are important enzymes involved in the metabolism of reactive oxygen species in the honeybee, and a reduction in the activity of these enzymes reduces cellular defenses against reactive oxygen species (ROS) and exacerbates oxidative damage [26,27]. After 48 h of low-temperature treatment (T2), we noticed a marked decrease in GSH, SOD, and CAT levels (*p* < 0.05; Figure 2). Moreover, GSH content was significantly reduced after 24 h of low-temperature treatment (T1), but SOD and CAT did not change significantly. These findings suggest that low-temperature stress triggers oxidative stress in honeybee new pupae brains, which leads to the consumption of antioxidant substances.

### 3.3. KEGG Pathway Altered Under Low-Temperature Stress

**Figure 3:**
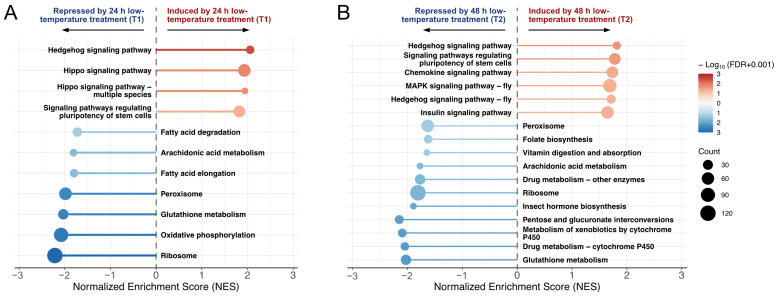
Plot of normalized enrichment score (NES) of Molecular Signature Database (MSD) CP:KEGG gene sets from Gene Set Enrichment Analysis (GSEA) for T1 vs. CK (**A**) and T2 vs. CK (**B**). Enriched pathways with FDR  <  0.05 are shown (red to blue coloring based on significance, with deeper red hues signifying increased pathway induction significance, and deeper blue hues denoting enhanced significance in pathway repression).

To explore the gene profile altered in pupae brains under low-temperature stress, and screen phenotype specific responsive genes, Gene Set Enrichment Analysis (GSEA) based on KEGG database was performed between the control group (CK) and treatment groups (T1, T2) (Figure 3A,B). A series of pathways was significantly altered after low-temperature treatment (FDR < 0.05); the hedgehog signaling pathway was significantly induced in both T1 and T2; and ribosome, peroxisome, glutathione metabolism, and arachidonic acid metabolism were inhibited in both T1 and T2. Interestingly, a few more metabolic pathways were inhibited after more intense low-temperature treatment, such as pentose and glucuronate interconversions, insect hormone biosynthesis, and folate biosynthesis.

**Figure 4:**
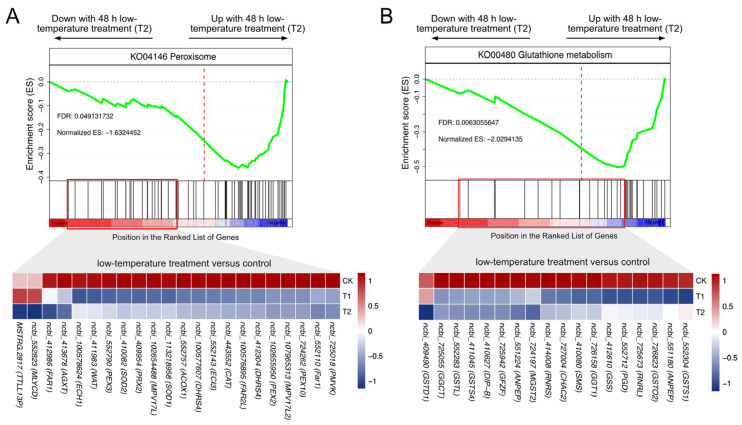
GSEA analysis revealed a significant enrichment of peroxisome pathway ((**A**), top) and glutathione metabolism ((**B**), top) pathway in 48 h low-temperature treatment (LTT) group (vs. control, T2); these pathways were repressed, and FDR values are shown. Heat-map illustrated the expression level of core-enrichment (leading-edge) genes of peroxisome pathway ((**A**), bottom) and glutathione metabolism pathway ((**B**), bottom) in 48 h LTT group (T2). The expression levels were normalized by row while simultaneously presenting the expression levels of the same gene under 24 h of LTT (T1).

A significant increase in oxidative stress was noted in pupal brains following 48 h of low-temperature treatment (referred to as T2 in Section 3.2). GSEA revealed that exposure to low temperatures significantly disrupted glutathione metabolism and peroxisome pathways (Figure 3A,B), which are known to play a critical role in the regulation of ROS metabolism. These pathways may be involved in the underlying regulatory mechanism of oxidative stress. Subsequently, we identified the core-enrichment (leading edge) genes associated with these pathways in the T2 vs. CK comparison using enrichment score screening to pinpoint the key gene set involved in this process (represented in Figure 4A,B).

### 3.4. Identification of Genes Expression Pattern Under Low-Temperature Stress

**Figure 5:**
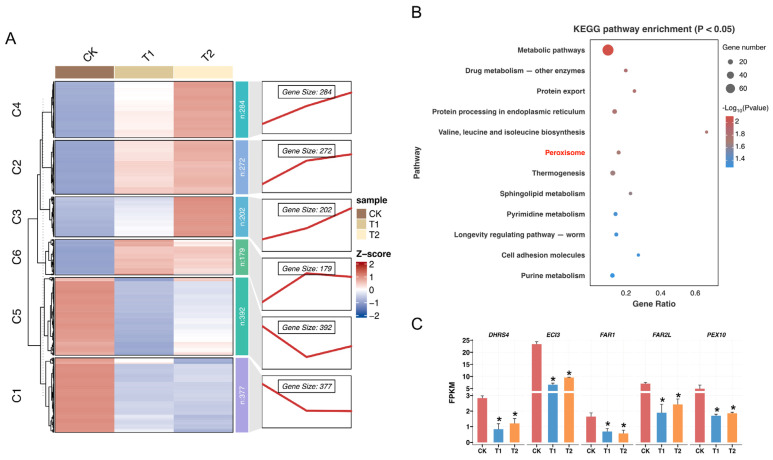
(**A**) Clustering analysis of differentially expressed genes (DEGs) in pupal brains *Apis mellifera* during low-temperature stress (LTS). In the left panel, heat maps illustrate DEGs expression profiles (RNA-Seq) of six soft clusters. The right panel displays the expression trends of DEGs throughout the LTS stages in each soft cluster. The Z-score indicates gene expression values normalized by column. (**B**) Enrichment analysis of KEGG pathways for DEGs in Cluster 1 and Cluster 5 displaying significantly enriched pathways (*p* < 0.05). The Peroxisome pathway in red highlights it is KEGG enriched in consistent with GSEA result in Figure 4A. (**C**) DEGs related to the peroxisome pathway display significant alterations after 24 h and 48 h of LTS. Asterisks indicate significant differences between comparison group (* *p* < 0.05, Student’s *t*-test. T1, T2 compared with the CK, respectively), as determined by RNA-seq analysis.

To gain insight into the patterns of gene regulation in the pupal brains under low temperatures, we performed expression trend analyses based on the fuzzy c-means clustering algorithm. A total of 1706 DEGs identified in the control group (CK) and two low-temperature treatments were categorized into six clusters. The determination of the cluster number was based on the inflection point obtained from the sum of squared changes computed from the expression matrix (Figure S1). Through hierarchical clustering, four upregulated expression patterns (clusters 2, 3, 4, and 6) and two downregulated expression patterns (clusters 1 and 5) were discerned (Figure 5, left panel). A total of 769 DEGs (377 in cluster 1 and 392 in cluster 5) displayed significant downregulation post-treatment, as shown in Figure 5A, right panel.

To determine the biological significance of DEGs in cluster 1 and cluster 5, which demonstrate marked repression in low-temperature environments, a KEGG enrichment analysis was executed. The analysis identified the enrichment of 13 pathways in DEGs within these clusters, particularly in the peroxisome pathways (*p* < 0.05) (Figure 5B). In addition, significant variances were detected in the expression levels of five genes related to peroxisome pathways following both 24 h and 48 h low-temperature treatments, including *PEX10*, a gene essential for the peroxisome function (Figure 5C).

### 3.5. RT-qPCR Verification

**Figure 6:**
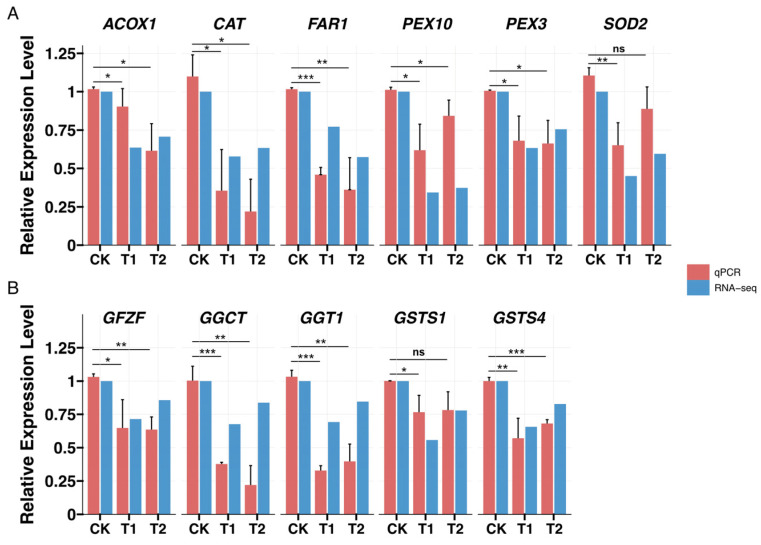
Verification of low-temperature stress on gene expression pattern in pupal brains *Apis mellifera* by RT-qPCR. (**A**) displays relative expression levels of six key genes in peroxisome pathway. (**B**) displays relative expression levels of five key genes in glutathione metabolism pathway. Data in the figure are mean + SD. Asterisks indicate significant differences between comparison group (* *p* < 0.05, ** *p* < 0.01, *** *p* < 0.001, ns *p* > 0.05, Student’s *t*-test, T1, T2 compared with the CK, respectively).

To validate the transcriptome findings, RT-qPCR analysis was performed on 11 core enriched genes found in the peroxisome pathway (Figure 6A) and glutathione metabolism pathway (Figure 6B). The results showed that the expression patterns of most genes were consistent with the transcriptome data, indicating their reliability.

## 4. Discussion

Extensive postembryonic neurogenesis occurs in the brain of the honeybee [28], and deviations from the optimal brood temperature (35 °C) have been shown in previous research to influence neural synapse numbers, memory formation, and dancing behavior in honeybees [29,30,31]. Our previously conducted research has indicated that low-temperature stress can have a two-fold effect on the neurological development of pupae in honeybees. Exposure to temperatures as low as 20 °C can result in developmental arrest throughout the pupal body, including the brain, and the accumulation of neurological damage. This damage is further evidenced by significant impairments in associative learning and memory abilities observed in honeybees that experienced low temperatures during their pupal stage [12]. However, there is insufficient definitive evidence to support the notion that exposure to low temperatures during developmental stages induces heightened oxidative stress in the honeybee pupal brains, a region predominantly housing the developing central nervous system. Therefore, this study investigated the alteration in antioxidant status in honeybee pupae brains, alongside an analysis of transcriptome sequencing data, to explore the underlying mechanism of induced ROS in the honeybee pupae nervous system.

### 4.1. Low-Temperature Stress Disrupts Oxidative Homeostasis in Honeybee Pupae Brains

Low temperature heightens oxidative stress in honeybee pupae brain. Cells in the human brain, comprising only 2% of body weight, consume approximately 20% of the body’s oxygen [32,33]. This makes the brain highly susceptible to the accumulation of ROS. Elevated levels of ROS produced during oxygen utilization are established contributors to neurotoxicity and degenerative processes seen in conditions such as Alzheimer’s and Parkinson’s [34,35]. In the context of insects, research indicates that unfavorable ambient temperatures can trigger ROS accumulation, leading to heightened oxidative stress in individuals [36,37,38,39]. Several studies have highlighted the significance of ROS-scavenging mechanisms in safeguarding insects against temperature-related stresses, involving various antioxidant compounds and enzymes [40,41,42]. Conversely, some studies have proposed that under low-temperature stress, the diminished efficacy of antioxidant enzymes can result in ROS buildup [43]. Through our investigation, we observed a marked decline in the concentration of GSH in the honeybee pupal brains, along with diminished SOD and CAT activities following 48 h of low-temperature stress (Figure 2). These results indicate the occurrence of moderate oxidative stress under conditions of prolonged exposure to severe cold.

### 4.2. Low-Temperature Stress Induces the Depletion of Antioxidant Compounds Through Suppression of Glutathione Metabolism Pathway

The nervous system relies on reduced glutathione (GSH) to maintain homeostasis and support metabolic processes [44]. GSH plays a crucial role in mitigating oxidative stress-induced neurotoxicity, as evidenced by the protective effects of GSH injections in honeybee antennal lobes against oxidative stress-related deficits in olfactory learning and memory [45]. A well-established GSH cycle is responsible for regulating GSH levels [46]. However, certain studies have indicated that prolonged exposure to cold temperatures may deplete the glutathione pool, leading to oxidative stress [47]. In this study, GSEA revealed that the glutathione metabolism pathway was significantly suppressed in both low-temperature groups (Figure 4B). RT-qPCR results showed that the expression of several genes encoding key enzymes of the glutathione metabolic pathway was significantly down-regulated under low-temperature treatment, which generally agreed with the findings from the RNA-seq analysis (Figure 6B), indicating the sequencing data were reliable. Glutathione (GSH) is produced through a two-step process, with the final step catalyzed by the enzyme glutathione synthetase (GSS) [48]. The downregulation of the gene *GSS* encoding this enzyme, as indicated by its core enrichment in our GSEA results (see Figure 4B), may impede the efficiency of GSH regeneration. Additionally, the regulation of the core-enriched gene *PGD*, identified through GSEA and responsible for encoding 6-phosphogluconate dehydrogenase, could potentially disrupt the transition from oxidized glutathione (GSSG) to GSH by affecting the NADP/NADPH cycle [49,50,51], thereby leading to GSH depletion. Overall, these findings provide support for the notion that low-temperature stress inhibits the glutathione metabolism pathway, leading to a blockade in the synthesis of the antioxidant GSH and an increase in oxidative stress in the pupal brain.

### 4.3. The Impairment of Peroxisome Function Under Low-Temperature Stress Could Be a Contributing Factor to the Accumulation of ROS

Peroxisomes, ubiquitous cell organelles, play a crucial role in both generating and scavenging ROS within the cell [52]. An imbalance between peroxisomal ROS production and elimination results in oxidative stress, linked to various human neurological disorders [53]. In insects, peroxisomes are implicated in responding to environmental stress across different developmental stages [54], yet their regulatory role in low-temperature neural toxicity during insect development remains insufficiently explored. Here, we reported that the import of peroxisomal matrix proteins and the proliferation of peroxisomes may be impeded by low temperatures, serving as a potential cause for the decreased antioxidant enzyme activity and heightened oxidative stress observed in the honeybee pupal brains under low-temperature exposure.

Under low-temperature stress conditions, there is an observed inhibition of the import pathway for peroxisomal matrix proteins, leading to a deficiency in peroxisomal hydrogen peroxide detoxification. Identification of the key gene *PEX10*, associated with the peroxisome pathway, revealed diminished expression levels under low-temperature stress through clustering analysis of DEGs (Figure 5C). GSEA demonstrated its core enrichment, as shown in Figure 4A. The downregulation of *PEX10* was further validated through RT-qPCR analysis in both low-temperature treatments (Figure 6A). The gene *PEX10* encodes peroxin 10, which plays a crucial role in the import of peroxisomal matrix proteins [55]. It is noteworthy that *PEX2*, the gene encoding peroxin 2, displayed core enrichment in the peroxisome pathway. Evidence suggests a synergistic function between peroxin 10 and peroxin 2 in the aforementioned process [56]. Because the dense peroxisomal matrix houses vital enzymes involved in detoxifying hydrogen peroxide [57], it is hypothesized that low-temperature stress inhibits the expression of *PEX10*, thereby leading to the suppression of peroxisomal hydrogen peroxide detoxification. This might explain the inhibited antioxidant enzyme activity in honeybee pupal brains at low temperatures in this study.

Low-temperature stress may also inhibit peroxisome proliferation. In response to changing environmental and physiological conditions, peroxisomes, as dynamic organelles, have the capacity to swiftly adjust their abundance. This adaptive capability enables them to control their proliferation, morphology, and intracellular mobility, as well as to interact with other organelles [58,59]. In the present study, the *PEX3* gene was identified by screening for core enrichment genes in the peroxisome pathway. The peroxisomal biogenesis factor 3 gene (*PEX3*) encodes the peroxin 3 protein, which acts as a key player in initiating membrane assembly in peroxisome biogenesis [60]. This protein is vital for the translocation of membrane proteins from the endoplasmic reticulum to the peroxisomal membrane, thereby contributing significantly to the development of the peroxisomal membrane [61,62,63]. The knockdown of *PEX3* leads to decreased peroxisome proliferation, causing the accumulation of cellular components and a notable reduction in cell proliferation [64]. RT-qPCR showed a significant suppression of *PEX3* in both low-temperature treatments; this might cause impeding peroxisome proliferation in the pupae brain, resulting in an insufficient ROS scavenging function. Additionally, there is evidence from some studies suggesting that a decrease in *PEX3* levels does not immediately cause a reduction in peroxisome numbers [65], possibly due to the extended half-life of peroxisomes [66]. During the current investigation, it was found that a period of 24 h under low-temperature stress did not induce significant modifications in antioxidant enzyme activities within the pupal brains. In contrast, the extended 48-h treatment resulted in noteworthy changes, indicating a potential delay in the inhibition of peroxisome proliferation, thereby affecting ROS regulation.

## 5. Conclusions

In the present study, we found that the inhibition of the glutathione and peroxisome pathways, along with the downregulation of key genes, resulted in a reduced ability to metabolize ROS, which increased oxidative stress levels in the pupal brain that underwent cold stress. The decline in pupae brain GSH content, along with the decreased activities of SOD and CAT enzymes, further bolsters this interpretation. The results provide a biochemical and molecular understanding for the impairment in honeybee neural system development due to low temperatures, which helps to explore avenues for mechanistic studies into cold-induced neural damage in insects.

## Data Availability

The datasets presented in this study can be found in online repositories. The names of the repository/repositories and accession number(s) can be found below: NCBI SRA database (accession number: PRJNA1221518).

## Supplementary Materials

The following supporting information can be downloaded at: https://www.mdpi.com/article/10.3390/insects16030250/s1, Table S1: Primers for RT-qPCR in present study; Table S2: Gene expression matrix obtained by RNA-seq; Table S3: The detail of KEGG pathways enriched by GSEA in the CK vs T1 group; Table S4: The detail of KEGG pathways enriched by GSEA in the CK vs. T2 group; Table S5: The clustered profiles analyzed by ClusterGVis; Figure S1: Optimal number of clusters.
