# Supplementary material for: Biochemical and Transcriptomic Analysis Reveals Low Temperature-Driven Oxidative Stress in Pupal Apis mellifera Neural System
Source: Insects. 2025 Mar 1;16(3):250. doi: 10.3390/insects16030250 (PMC11942804; doi:10.3390/insects16030250)
Supplement: Supplementary file 1 [file insects-16-00250-s001.zip › insects-3383298-SI-Table S1, Figure S1.pdf]

Table S1. Primers for RT-qPCR in present study.

Gene	Gene ID	Primer sequence
ACTIN	LOC413144	F: TGCCAACACTGTCCTTTCTG R: AGAATTGACCCACCAATCCA
Peroxisome pathway		
PEX10	LOC724262	F: GCAAGCCAAGCCGAAATTCTAC R: CTTCACCCAACGTTTGATTGCC
PEX3	LOC552790	F: ATGTTTTCAAGACTTCGTGGATTTGTG R: CGACGTTTTGATCTGTCCAGC
SOD2	LOC410082	F: CATTCACGAAGGTGTATAGTGTATCTG R: GCATAATTTCGGCGGAAATAATAGGTTCC
CAT	LOC443552	F: CGAAATTGAAGACGATACGATGACTG R: CATGGACTACACGTTCCGGAATC
FAR1	LOC412986	F: CACCAAACTAGTTTAATCAGATTGGAAGG R: CCTTTTCGGCCGTATCAAAACATAG
ACOX1	LOC552757	F: CTGTGTTGACTTTCATCAACTGCC R: CTCGTTCGCGACGTTCTATTG
Glutathione pathway		
GSTS1	LOC552304	F: ACATTAACACGTTCCAACGCA R: GCGATCAAGCGAGAAATAGCC
GSTS4	LOC411045	F: CACAGATTAATGACAATGGCGAGTTAC R: CCAAGCAGATTAAACTGTTTGGCC
GSTD1	LOC409490	F: GCTGATTTTGCCATCCACACGA R: GTCATCAATCGTGGGTATCGTG
GFZF	LOC425942	F: GGAAAACAAGAGAAAGGAACTTTGTGC R: CAAGCAAGAGATGGTGGTCCATC
GGT1	LOC726158	F: GCTGTTCATCTTCGGACGATGC R: CTGACGATGTTCGTCAGTACTAGC
GGCT	LOC725055	F: CTACCAATACACAGAAGACCTTCACC R: TAGAGAATGATCCTCTTGTCCAGAAAC

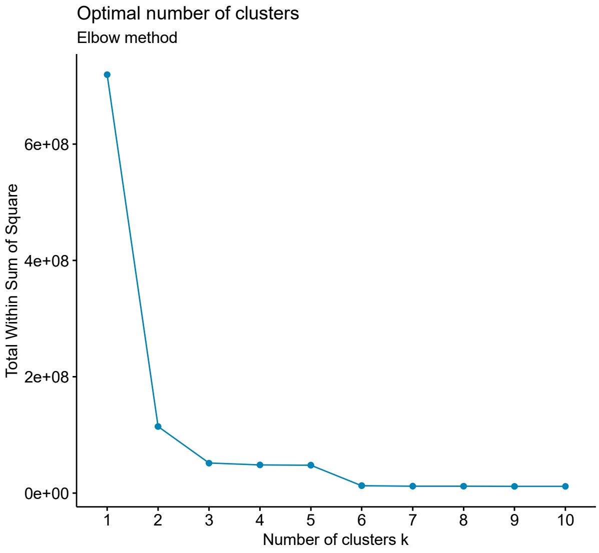

Figure S1. Optimal number of clusters.
